# Sb_2_Te_3_ nanoparticle-containing single-walled carbon nanotube films coated with Sb_2_Te_3_ electrodeposited layers for thermoelectric applications

**DOI:** 10.1038/s41598-023-33022-4

**Published:** 2023-04-08

**Authors:** Rikuo Eguchi, Koki Hoshino, Masayuki Takashiri

**Affiliations:** Department of Materials Science, Tokai University, Hiratsuka, Kanagawa 259-1292 Japan

**Keywords:** Thermoelectrics, Carbon nanotubes and fullerenes, Electrochemistry

## Abstract

Single-walled carbon nanotubes (SWCNTs) are promising thermoelectric materials owing to their flexibility and excellent durability when exposed to heat and chemicals. Thus, they are expected to be used in power supplies for various sensors. However, their thermoelectric performances are inferior to those of inorganic thermoelectric materials. To improve the thermoelectric performance while maintaining the excellent characteristics of SWCNTs, a novel approach to form inorganic thermoelectric layers on the SWCNT bundle surfaces using electrodeposition is proposed. We synthesized Sb_2_Te_3_ nanoparticle-containing SWCNT films and coated them with electrodeposited Sb_2_Te_3_ layers. The Sb_2_Te_3_ nanoparticles were synthesized via a spontaneous redox reaction, which were then added to a SWCNT dispersion solution, and films were produced via vacuum filtration. At higher nanoparticle contents in the films, the Sb_2_Te_3_ electrodeposited layers completely covered the SWCNT bundles owing to the increase in the concentration of precursor ions near the SWCNT bundle surface, which in turn was the result of melted nanoparticles. The thermoelectric performance improved, and the maximum power factor at approximately 25 °C was 59.5 µW/(m K^2^), which was 4.7 times higher than that of the normal SWCNT film. These findings provide valuable insights for designing and fabricating high-performance flexible thermoelectric materials.

## Introduction

Thermoelectric generators are promising energy harvesting devices. They produce electrical energy via carrier diffusion in response to heat flux produced by a temperature gradient in thermoelectric materials. The widespread development of Internet of Things (IoT) has facilitated the emergence of the flexible thermoelectric generator technology^1–4^. This is because the IoT technology requires a large number of sensors, and wireless power supplies for the sensors using ambient heat sources are indispensable for the technology. In addition, the flexibility favors the installation of generators in a variety of heat sources, such as human bodies and curved surface objects^5–8^.

Generally, flexible thermoelectric materials include conductive organic materials^9–11^, single-walled carbon nanotubes (SWCNTs)^12–14^, and their composites^15–17^. Among them, SWCNTs are excellent as power supplies for IoT sensors because they exhibit excellent heat and chemical durability. Although SWCNTs demonstrate excellent characteristics for flexible thermoelectric generators, their thermoelectric performances are inferior to those of inorganic chalcogenides such as Bi_2_Te_3_-based alloys^18^. The thermoelectric performance is expressed by the dimensionless figure-of-merit *ZT* = *σS*^2^*T*/*κ* and power factor *PF* = *σS*^2^, where *σ*, *S*, *T*, and *κ* represent the electrical conductivity, Seebeck coefficient, absolute temperature, and thermal conductivity, respectively.

To increase the thermoelectric performance, an effective approach is to combine the SWCNTs with inorganic thermoelectric materials. Jin et al. used a sputtering technique to fabricate a flexible thermoelectric material comprising highly ordered bismuth telluride (Bi_2_Te_3_) nanocrystals anchored on a single-walled carbon nanotube network; this material exhibited a high Seebeck coefficient^19^. Wu et al.^20^ prepared hybrid thin films of p-type SWCNTs and antimony telluride (Sb_2_Te_3_) nanoplates, which exhibited high power factors, through the combination of vacuum filtration and annealing. In addition to these studies, excellent thermoelectric materials have been proposed by combining CNTs with inorganic and organic compounds^21,22^. In our previous study, we prepared a SWCNT dispersion solution in Bi_2_Te_3_ nanoplates using a solvothermal synthesis and produced flexible films through drop-casting, resulting in an increase in the thermoelectric performance^23–25^.

These pioneering studies motivate us to further increase the thermoelectric performance by combining the SWCNTs and inorganic chalcogenides. A favorable approach is to coat the SWCNT bundle surface with an inorganic chalcogenide via electrodeposition^26–28^. As electrodeposition is a wet process, not only the surface of the SWCNT film but also the inside of film can be electrodeposited by the electrolyte penetrating the gaps in the film. However, it is challenging to effectively deposit the inorganic layer on the SWCNT bundle surface^29^. This is because the SWCNT films in the bundle form exhibited low electrical conductivity compared to metals^30–32^, and several precursor ions in the electrolyte were not attracted to the SWCNT bundle surface. Therefore, it is necessary to explore different methods for performing electrodeposition on the SWCNT bundle surface^33^.

In this study, a novel approach was performed to increase the thermoelectric performance of the flexible SWCNT films. We mixed Sb_2_Te_3_ nanoparticles with an SWCNT dispersion solution; subsequently, we prepared the p-type films via the drop-casting method. Sb_2_Te_3_ is a traditional p-type chalcogenide, which exhibits high thermoelectric performance near 300 K^34–36^, and Sb_2_Te_3_ nanoparticles were synthesized via a spontaneous redox reaction^37^. This method uses pitting corrosion, which is caused by the action of chloride ions on the substrate surface, as well as galvanic replacement, which is caused by the difference in the standard reduction potential between the materials. The p-type Sb_2_Te_3_ layers were formed on the nanocomposite films via the electrodeposition method^38^. We tuned the quantity of Sb_2_Te_3_ nanoparticles in the SWCNT films to investigate the films with high thermoelectric performance.

## Experimental setup

**Figure 1:**
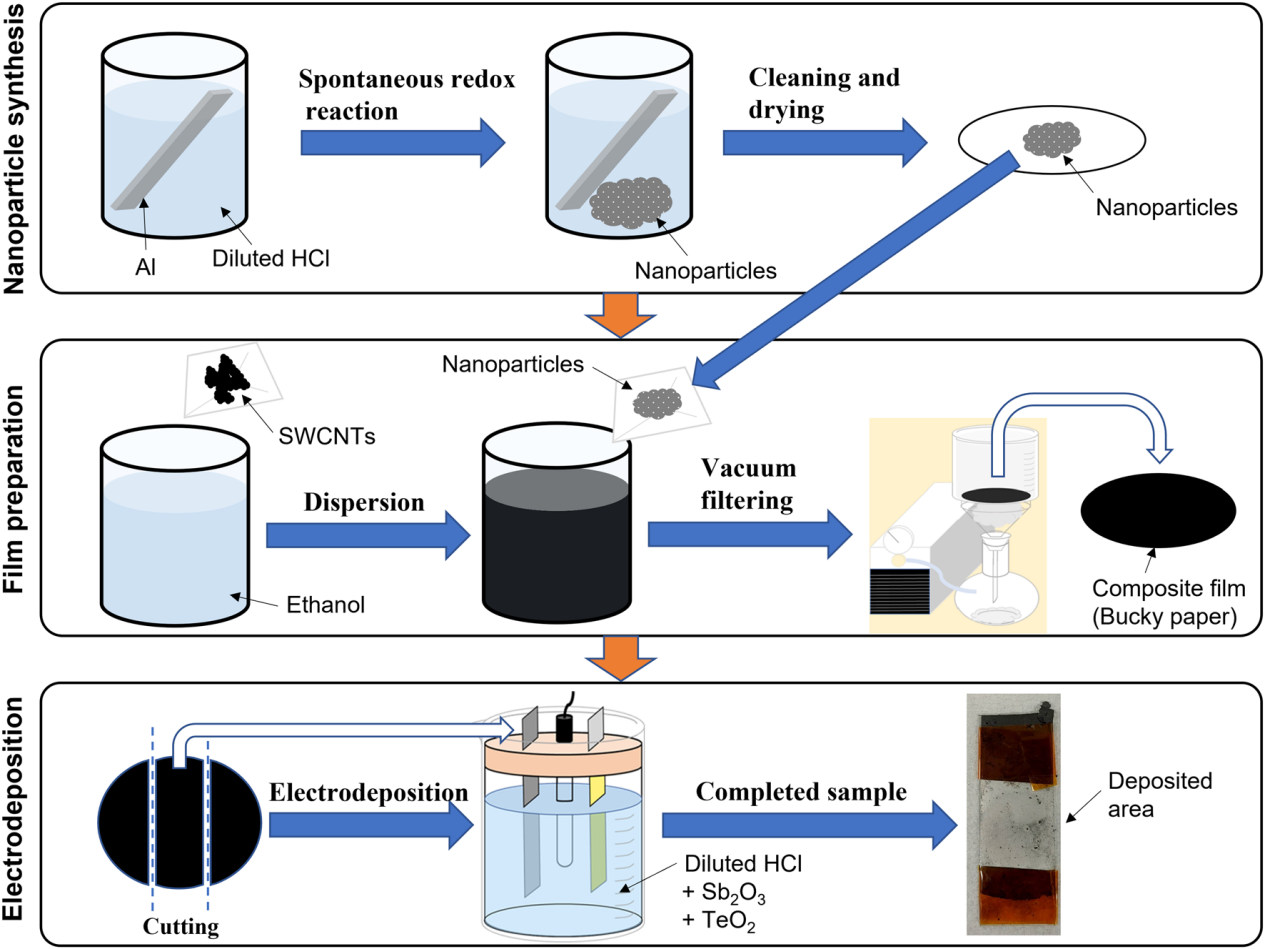
Schematic diagram of the fabrication processes: nanoparticle synthesis, nanocomposite film preparation, and electrodeposition.

Figure 1 shows a schematic of the fabrication process of the SWCNT films containing Sb_2_Te_3_ nanoparticles and the coating of the Sb_2_Te_3_ electrodeposited layers. First, the nanoparticles were synthesized via the spontaneous redox reaction using an aluminum substrate (50 mm × 100 mm, 2 mm thick). Prior to nanoparticle synthesis, to remove any oxide layers from the aluminum substrate, it was placed in a 1 M NaOH (Fujifilm Wako Pure Chemical Co.) solution for 5 min, followed by cleaning in deionized (DI) water (i.e., > 18 MΩ). The spontaneous redox reaction was conducted between the sacrificial aluminum plate and an electrolyte containing 0.02 M Sb_2_O_3_ (Kojundo Chemical Laboratory Co., Ltd.), 0.04 M TeO_2_ (Kojundo Chemical Laboratory Co., Ltd.), and 4.0 M HCl (Fujifilm Wako Pure Chemical Co.) for 40 min at approximately 25 °C. After the reaction, the nanoparticles were washed with DI water, filtered, and dried under vacuum at 60 °C for 12 h.

For film preparation, SWCNTs synthesized by the super-growth method (SG-CNTs) (ZEONANO SG101, ZEON Co.) were used as the starting material^39^. Powdered SG-CNTs (80 mg) were dispersed in 40 mL of ethanol to prepare an SWCNT dispersion solution with a concentration of 0.25 wt%, and homogenized mixing was ensured using an ultrasonic homogenizer (SONICS 85, AZONE Co.). Next, the fabricated nanoparticles (5, 10, 50, and 100 mg) were added to the SWCNT dispersion solution, followed by mixing using the ultrasonic homogenizer. The nanocomposite films were prepared by a vacuum filtration method. A membrane filter (PTFE, 90 mm diameter: ADVANTEC) was placed in a filter holder in a suction bottle, and the dispersion solution was filtered by reducing the pressure in the suction bottle using a rotary pump. The entire volume of the nanoparticle-containing SWCNT-dispersion solution was poured dropwise onto the filter and aspirated for 1 h to produce the nanocomposite films with a diameter of 80 mm. After drying for air-drying 24 h, the nanocomposite films (thickness = 100 µm) were removed from the membrane filter.

For electrodeposition, Sb_2_Te_3_ layers were prepared at approximately 25 °C by potentiostatic electrodeposition using a standard three-electrode cell. The electrolytes contained mixtures of 1.88 mM Sb_2_O_3_, 0.63 mM TeO_2_, and 0.58 M HCl diluted with DI water. The working electrode (electrode area: 7.8 cm^2^) was the nanocomposite film, which was fixed to a stainless-steel plate using polyimide tape. A platinum-coated titanium mesh on a titanium plate was used as the counter electrode (electrode area: 7.8 cm^2^). A Ag/AgCl (saturated KCl) electrode was used as the reference electrode. The electrode voltage was set at − 0.01 V using a potentiostat/galvanostat (HA-151B, Hokuto Denko) based on our previous report^40^, and electrodeposition was performed for 1 h.

The precise structure of the nanoparticles was analyzed using high-resolution transmission electron microscopy (HR-TEM, JEOL JEM-ARM200F), selected area electron diffraction (SAED), and energy-dispersive X-ray spectroscopy (EDX) elemental mapping. The crystallographic properties of the nanoparticles and films were evaluated by X-ray diffraction (XRD; Mini Flex II, Rigaku) using Cu-Kα radiation (λ = 0.154 nm in the 2*θ* range from 20° to 70°). The microstructures of the films were analyzed with field emission scanning electron microscopy (FE-SEM, Hitachi S-4800). The chemical compositions of the nanoparticles and nanocomposite films were determined with an electron probe microanalyzer (EPMA, Shimadzu EPMA-1610). The compositions of the samples were calibrated using the ZAF4 program installed with the EPMA-1610.

The in-plane electrical conductivity was measured at approximately 25 °C by a four-point probe method with an accuracy of ± 5%. The in-plane Seebeck coefficient of the nanocomposite films was measured at approximately 25 °C with an accuracy of ± 7%^41^. We used two K-type thermocouples of 0.1 mm in diameter, which were pressed on the center of the film. The distance between the thermocouples was 13 mm. One end of the film was connected to a heat sink and the other end to a heater. The Seebeck coefficient was determined as the ratio of the potential difference (Δ*V*) along the film to the temperature difference. The power factor was estimated from the measured electrical conductivity and Seebeck coefficient with an accuracy of ± 10%.

## Results and discussion

### Synthesis of the Sb_2_Te_3_ nanoparticles

The Sb_2_Te_3_ nanoparticles were synthesized via a spontaneous redox reaction, where an aluminum plate was immersed in a hydrochloric acid solution containing HTeO_2_^+^ and SbO^+^ ions. The difference in the redox potential for Al^3+^/Al^0^ (*E*^0^ =  − 1.676 V vs. NHE) is more cathodic than for SbO^+^/Sb^0^ (*E*^0^ =  + 0.212 V vs. NHE) and HTeO_2_^+^/Te^0^ (*E*^0^ =  + 0.353 V vs. NHE)^42^. The galvanic displacement of the aluminum plate to the Sb_2_Te_3_ nanoparticles can be represented as follows:

1 $$\begin{aligned} & {\text{3HTeO}}_{{2}}^{ + } \left( {{\text{aq}}} \right) \, + {\text{ 2SbO}}^{ + } \left( {{\text{aq}}} \right) \, + {\text{ 13H}}^{ + } \left( {{\text{aq}}} \right) \, + {\text{ 6Al}}^{0} \left( {\text{s}} \right) \, \to \\ & {\text{Sb}}_{{2}} {\text{Te}}_{{3}}^{0} \left( {\text{s}} \right) \, + {\text{ 8H}}_{{2}} {\text{O }} + {\text{ 6Al}}^{{{3} + }} \\ \end{aligned}$$

**Figure 2:**
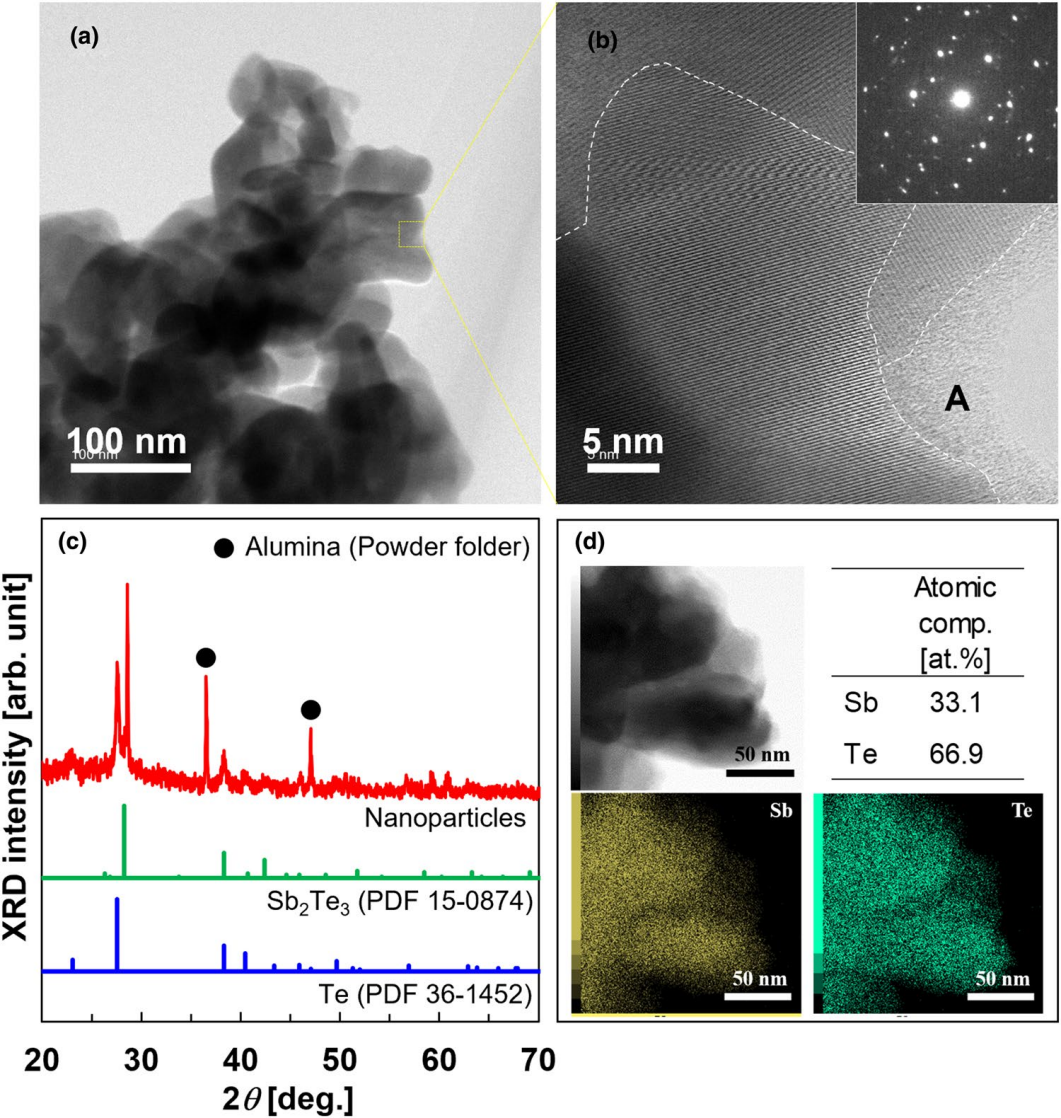
TEM images of Sb_2_Te_3_ nanoparticles prepared via spontaneous redox reaction. (**a**) Typical Sb_2_Te_3_ nanoparticles, (**b**) corresponding SAED pattern and HR-TEM image of the nanoparticle in (**a**), (**c**) XRD pattern of the nanoparticles, and (**d**) atomic composition and elemental mapping.

Note that the direct formation of the Sb_2_Te_3_ intermetallic compound is thermodynamically favorable over the formation of Sb^0^ and Te^0^ because the Gibbs free energy of Sb_2_Te_3_ formation is negative (i.e. Δ*G*_f_^0^ =  − 57.5 kL/mol)^42^. In Fig. 2a, the TEM micrograph shows that irregularly shaped particles with a size less than 100 nm are agglomerated. In Fig. 2b, the HR-TEM image of the near-surface of the nanoparticles shows that the nanoparticles exhibit a polycrystalline phase with grain boundaries of nanometer-sized single crystals, which corresponds to the SAED pattern in the inset. No lattice fringes are observed on the topmost surface of the particles (area A), indicating the formation of an amorphous layer. In Fig. 2c, the XRD pattern of the nanoparticles includes the peaks originating from Sb_2_Te_3_ and Te, corresponding to the SAED pattern in the inset of Fig. 2b. The elemental mapping and atomic composition of the nanoparticles are shown in Fig. 2d. In the elemental mapping, antimony and tellurium are almost uniformly distributed within the particles. The atomic percentages of antimony and tellurium are 33.1 and 66.9 at%, respectively. This atomic composition ratio deviates from the stoichiometric ratio (Sb/Te = 40/60) by approximately 7% towards the Te-rich side because of the presence of a Te crystalline phase in addition to the Sb_2_Te_3_ crystalline phase.

For comparison, we synthesized Sb_2_Te_3_ nanoparticles via a spontaneous redox reaction using nickel and copper plates. The characteristics of the synthesized nanoparticles are provided in Supplementary Information (Fig. S1). As a result, the atomic compositions of the nanoparticles from the nickel and copper plates determined by EPMA significantly differ from the stoichiometric proportions: Sb/Te = 3.2/96.8 at% for the nickel plate and Sb/Te = 12.2/87.8 at% for the copper plate. This is because nickel and copper are less prone to pitting corrosion by chloride ions^43,44^, and thus the galvanic displacement reaction is not active.

### Formation of the nanocomposite films with electrodeposition

**Figure 3:**
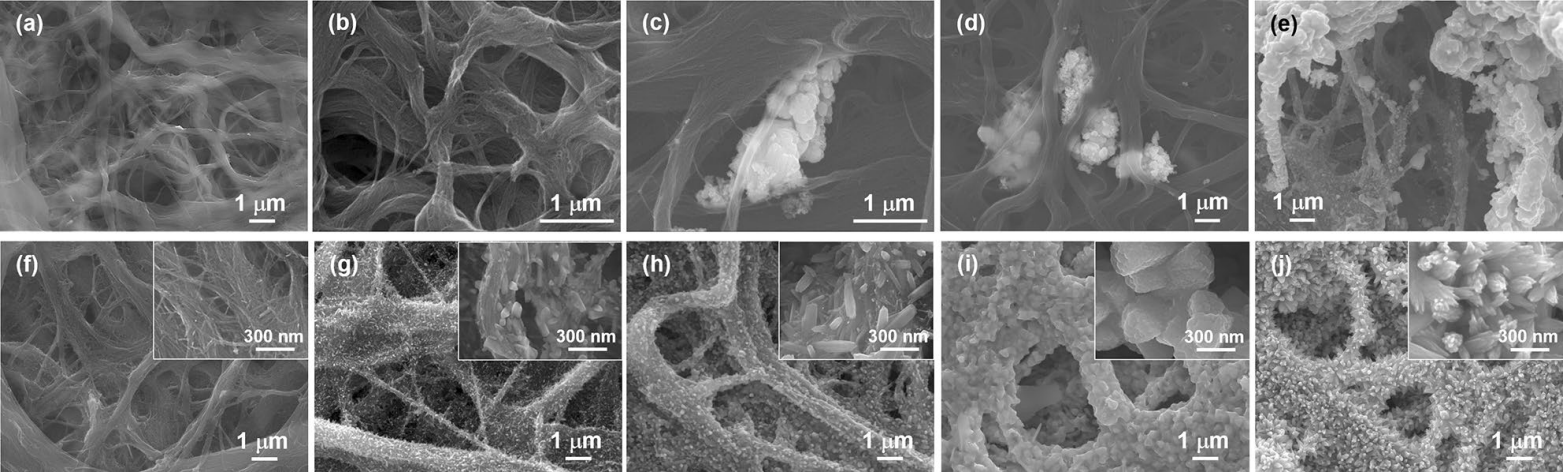
Surface SEM images of SWCNT films with different quantities of nanoparticles and excluding electrodeposition: (**a**) no nanoparticles, (**b**) 5 mg, (**c**) 10 mg, (**d**) 50 mg, and (**e**) 100 mg. Surface SEM images of SWCNT films with different quantities of nanoparticles and with electrodeposition: (**f**) no nanoparticles, (**g**) 5 mg, (**f**) 10 mg, (**i**) 50 mg, and (**j**) 100 mg.

Figure 3 shows the SEM images of the nanocomposite films without and with the nanoparticles. The SWCNT films without the nanoparticles are composed of entangled SWCNT bundles of different diameters (Fig. 3a). At 5 mg nanoparticles, the nanoparticles are not visible in the analysis area (Fig. 3b). However, at 10 mg, aggregated nanoparticles trapped between the SWCNT bundles are observed (Fig. 3c). Further increasing the nanoparticle content increases the size of the nanoparticle aggregates (Fig. 3d,e). Figure 3f–j show the electrodeposition performed on nanocomposite films of varying nanoparticle content (0–100 mg, respectively). In Fig. 3f, there are no Sb_2_Te_3_ layers on the bundle surface of the film, even though electrodeposition was performed. Therefore, in the absence of nanoparticles within the films, it is difficult to form an electrodeposited layer on the films. At 5 mg nanoparticles (Fig. 3g), tiny spines appeared on the SWCNT bundle surface. The enlarged image in the inset shows that angular crystals sparsely grew on the surface. At 10 mg nanoparticle content, the size and density of the angular crystals increase (Fig. 3h). At 50 mg, the crystals further grow and completely cover the SWCNT bundle surface (Fig. 3i), while at 100 mg, the crystal shape changes to a fine flower-like structure (Fig. 3j), indicative of dendritic growth. The electrodeposition layers are formed not only on the surface of the SWCNT film, but also on the inside of the film.

Table 1 shows the atomic compositions of the nanocomposite films with varying nanoparticle content after electrodeposition. Antimony and tellurium are not detected in the film without the nanoparticles. At a nanoparticle content of 5–100 mg, the atomic composition of the nanocomposite films is almost constant at Sb: 24 ± 1 at% and Te: 76 ± 1 at%, despite the large differences in crystal size and shape of the films. This is because the atomic composition of alloy films mainly depends on the applied voltage during electrodeposition^45,46^. Therefore, even though the atomic composition of the films deviated from the stoichiometric ratio (Sb: 40 at% and Te: 60 at%), an electrodeposition layer was formed on the surface of the SWCNT bundle by adding nanoparticles to the film. Further, the structure of the electrodeposited layer was tuned by changing the nanoparticle content. To evaluate the compositional homogeneity of the films, EDS maps are provided in Supplementary Information (Fig. S2). The EDS maps showed that antimony and tellurium were uniformly deposited on the surface of the SWCNTs and that the SWCNTs were less exposed. In addition, the XRD pattern of the typical nanocomposite film containing 10 mg of nanoparticles with electrodeposition is provided in Supplementary Information (Fig. S3).

**Table 1:** Atomic composition of SWCNT films with nanoparticles, followed by electrodeposition.

Quantity of nanoparticle [mg]	Atomic composition of composite film	
	Sb [at%]	Te [at%]
0	N/A	N/A
5	23.2	76.8
10	24.0	76.0
50	24.3	75.7
100	24.1	75.9

Here, we discuss the mechanism of crystal growth of the electrodeposited layers on the nanoparticle-containing SWCNT films. In previous reports, Bi_2_Te_3_ crystals were grown on CNT surfaces via the sputtering process and solvothermal synthesis^19,24^. In addition, thin layers were electrodeposited on a SWCNT surface by tuning the surface condition of the SWCNT films^29^. These results suggest that layers of inorganic chalcogenides can grow on the CNT surface via electrodeposition under appropriate conditions. The key factors of electrodeposition are the ion concentration around the SWCNTs and current density^47^. The quantity of chemicals used during electrodeposition was the same in all the samples; however, the concentration of the precursor ions around the SWCNTs increases as the nanoparticle content in the film increases because the nanoparticles dissolve when exposed to dilute HCl. As a result, the current density increases as shown in Supplementary Information (Table S1). Therefore, in the case of the SWCNT film without nanoparticles, Sb_2_Te_3_ crystals do not grow because the densities of the Sb and Te nuclei are too low, and the crystals possibly re-melt before the crystal size reaches the critical nucleus radius^48^. In the presence of nanoparticles, the ion concentration around the SWCNTs and current density passing through the film increase and promote crystal growth. However, when the ion concentration and current density are too high, dendritic growth occurs^47^, as shown in Fig. 3j.

### Thermoelectric properties of nanocomposite films

**Figure 4:**
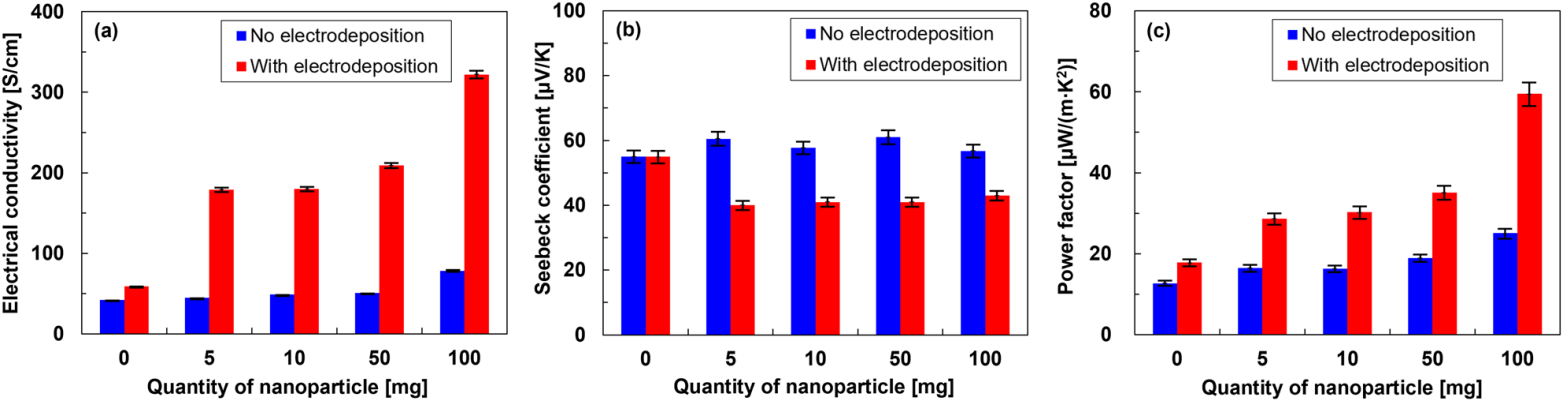
In-plane thermoelectric properties of SWCNT films with different quantities of nanoparticles and with electrodeposition and excluding electrodeposition. (**a**) Electrical conductivity, (**b**) Seebeck coefficient, and (**c**) power factor.

Figure 4 shows the in-plane thermoelectric properties of the nanocomposite films as a function of nanoparticle content. In Fig. 4a, the electrical conductivity of the normal SWCNT film (without nanoparticles and no electrodeposition) is 42 S/cm. As the nanoparticle content increases, the electrical conductivity of the nanocomposite film excluding electrodeposition slightly increases, indicating that the nanoparticles fill the gaps between the SWCNT bundles. The electrical conductivity of the nanocomposite film with 100 mg nanoparticles is 78 S/cm, which is 86% higher than that of the normal SWCNT film. The increase in electrical conductivity is due to the nanoparticles between the SWCNT bundles serving to increase the current paths. On the other hand, by performing electrodeposition on the films containing the nanoparticles, the electrical conductivity of the films is significantly affected by the quantity of nanoparticle. At 5 mg nanoparticle content and electrodeposition, the electrical conductivity is 179 S/cm, which is three times higher than that of the electrodeposited film without nanoparticles. At a higher nanoparticle content, the electrical conductivity further increases. At 100 mg, the electrical conductivity reaches 322 S/cm, which is 5.6 times higher than that of electrodeposited SWCNT film without nanoparticles. Therefore, the electrical conductivity is significantly increased by the increased thickness of the electrodeposited layer due to the increased quantity of nanoparticles.

In Fig. 4b, all films exhibit a p-type property due to the positive Seebeck coefficient. The Seebeck coefficient of the nanocomposite film excluding electrodeposition ranges from 55 to 60 µV/K and is independent of the nanoparticle content. On the other hand, by performing electrodeposition on the films containing the nanoparticles, the Seebeck coefficient of the films decreases. At 5 mg nanoparticle content, the electrodeposited film exhibits a Seebeck coefficient of 40 µV/K, which approximately remains the same even at higher nanoparticle contents. The decrease in the Seebeck coefficient compared to the normal SWCNT films occurs because the electrodeposited Sb_2_Te_3_ layer exhibits a low Seebeck coefficient owing to the deviation from the stoichiometric proportion^38^. Since the deviation from the stoichiometric ratio is constant regardless of the quantity of nanoparticles, the Seebeck coefficient is largely constant, while there are differences in the thickness and surface morphology of the electrodeposited layers.

In Fig. 4c, when the electrodeposition is not performed, the power factor slightly increases as the quantity of nanoparticles increases. The power factor of the nanocomposite film with 100 mg nanoparticles is 25.1 µW/(m K^2^), which is two times higher than that of the normal SWCNT film. On the other hand, when electrodeposition is performed on films containing the nanoparticles, the power factor of the films greatly increases. In particular, at 100 mg nanoparticles with electrodeposition, the power factor is 59.5 µW/(m K^2^), which is 2.4 and 4.7 times higher than that of the corresponding nanocomposite film with no electrodeposition and the normal SWCNT film, respectively. Here, we compared the power factor in this study with that shown in the above-mentioned literature, where hybrid thin films of SWCNTs and Sb_2_Te_3_ nanoplates were prepared by vacuum filtration, followed by annealing^20^. Although no annealing was performed, the maximum power factor in this study (59.5 µW/(m K^2^)) was comparable to the maximum value in the literature (55 µW/(m K^2^)).

Therefore, a significant improvement in the thermoelectric performance of Sb_2_Te_3_ nanoparticle-containing flexible SWCNT films subjected to Sb_2_Te_3_ layer electrodeposition is observed. To further improve the performance of the nanocomposite films, the Seebeck coefficient should be increased. A feasible approach is to optimize electrodeposition to form Sb_2_Te_3_ layers of stoichiometric proportion. In this study, the thermal conductivity of the nanocomposite films was not measured, and thus we calculated the thermal conductivity of the nanocomposite films based on the respective thermal conductivities of the SWCNT films, Sb_2_Te_3_ nanoparticles, and Sb_2_Te_3_ electrodeposited films. The calculated thermal conductivity is provided in Supplementary Information (Table S2). In the future, the calculated thermal conductivity should be verified by measuring the thermal conductivity of the nanocomposite films, and the thermoelectric properties should be analyzed in more detail.

## Conclusions

Herein, the thermoelectric performance of the flexible SWCNT films was improved by containing the Sb_2_Te_3_ nanoparticles synthesized via spontaneous redox reaction in the SWCNT films, which was followed by the electrodeposition of the Sb_2_Te_3_ layer on the SWCNT bundle surface. When the nanoparticles were not contained in the SWCNT film, the Sb_2_Te_3_ layers were not formed using the electrodeposition. In contrast, the nanoparticles were contained in the SWCNT films, and the Sb_2_Te_3_ crystals were grown on the SWCNT bundle surface, even though the atomic composition of the electrodeposited layer deviated from the stoichiometric proportion. The crystal size and density of the electrodeposited Sb_2_Te_3_ increased with an increase in the quantity nanoparticles. Furthermore, increasing the concentration of precursor ions around SWCNTs via nanoparticle melting was the key to crystal growth, thus leading to the increase in the current density. The maximum power factor of the SWCNT films with Sb_2_Te_3_ electrodeposition was 59.5 µW/(m K^2^), which was 4.7 times higher than that of the normal SWCNT film. The proposed method can be extended to various other chalcogenide-SWCNT materials, thus demonstrating considerable potential for fabricating thermoelectric generators using both p- and n-type flexible thermoelectric films with excellent performance.

## Supplementary Information

Supplementary Information.

## Data Availability

The authors declare that most data supporting the findings of this study are available within the paper and its supplementary information files. The rest of the data generated during and/or analyzed during the current study are available from the corresponding author upon reasonable request.
