# Supplementary material for: Sb2Te3 nanoparticle-containing single-walled carbon nanotube films coated with Sb2Te3 electrodeposited layers for thermoelectric applications
Source: Sci Rep. 2023 Apr 8;13:5783. doi: 10.1038/s41598-023-33022-4 (PMC10082793; doi:10.1038/s41598-023-33022-4)
Supplement: Supplementary file 1 — Supplementary Information. [file 41598_2023_33022_MOESM1_ESM.docx]

**Supplemental information**

**Sb_2_Te_3_ nanoparticle-containing single-walled carbon nanotube films coated with Sb_2_Te_3_ electrodeposited layers for thermoelectric applications**

Rikuo Eguchi, Koki Hoshino, Masayuki Takashiri^*^

*Department of Materials Science, Tokai University, Hiratsuka, Kanagawa 259-1292, Japan*

*Corresponding author. E-mail address: takashiri@tokai-u.jp (M. Takashiri).

The nanoparticles were synthesized via a spontaneous redox reaction using nickel and copper plates. Figure S1 shows the structural characteristics of the synthesized nanoparticles. The nanoparticles synthesized using the nickel plate (Fig. S1(a)) were grown with a dendritic structure. The atomic composition was Sb:Te = 3.2 at%:96.8 at%, which was significantly different from the stoichiometric proportion, Sb:Te = 40 at%:60 at%. The nanoparticles synthesized using the copper plate (Fig. S1(b)) were grown with a spherical structure. The atomic composition was Sb:Te = 12.2 at%:87.8 at%. These phenomena occurred because nickel and copper are less prone to pitting corrosion by chloride ion; thus, the galvanic displacement reaction was not active.

Figure S1. SEM images of nanoparticles prepared via spontaneous redox reaction using a (a) nickel plate and (b) copper plate. Insets show the atomic compositions of antimony and tellurium.


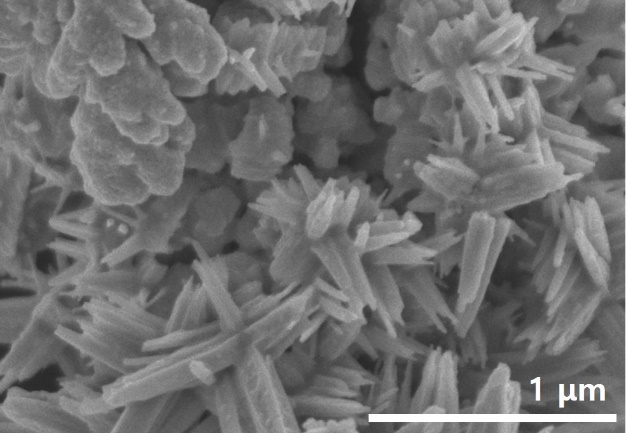

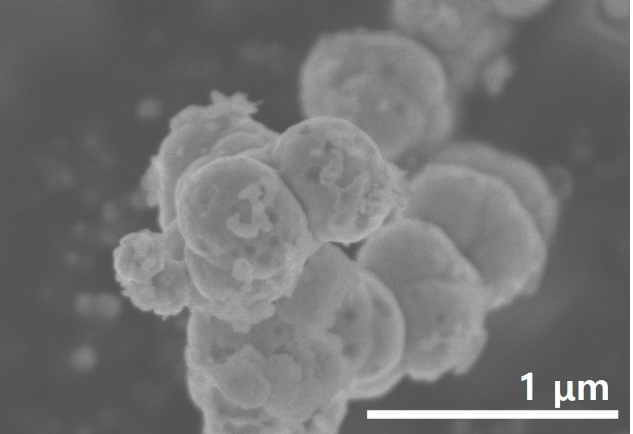


**(a)**

**(b)**

Table S1. Current densities observed during the electrodeposition of Sb_2_Te_3_ layers on SWCNT bundles with different quantities of nanoparticles in the films.

As observed in Table S1, the current density during electrodeposition increased as the quantity of nanoparticles increased. The increase in current density contributed to the formation of electrodeposition layers on SWCNT bundles.


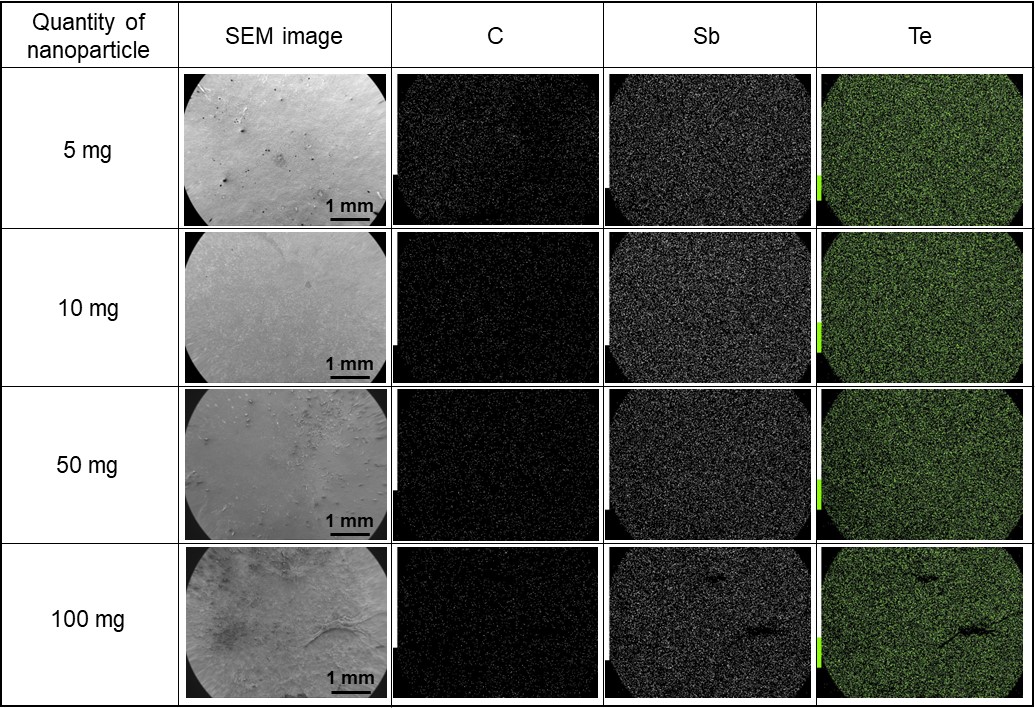
As observed in Fig. S2, the EDS maps showed that antimony and tellurium were uniformly deposited on the surface of the SWCNTs and that the SWCNTs were less exposed.

Figure S2. EDS maps of nanocomposite films with electrodeposition.


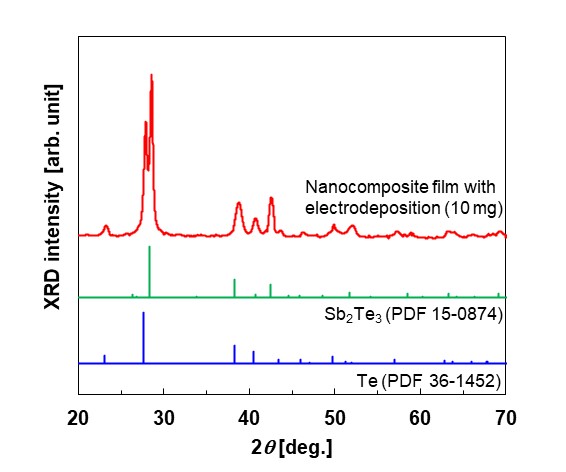


Figure S3. XRD pattern of the typical nanocomposite film with electrodeposition (10 mg nanoparticles).

As observed in Fig. S3, the XRD pattern of the nanocomposite film contained the peaks originating from Sb_2_Te_3_ and Te, which corresponded to those of the nanoparticles as shown in Fig. 2(c). Therefore, the crystalline phases of the nanocomposite film reflected those of the nanoparticles.

Table S2. Calculated thermal conductivity of nanocomposite films.

The thermal conductivity of the nanocomposite films was calculated based on the respective thermal conductivities of the SWCNT films (*κ* = 5.4 W/(m·K)) [1], Sb_2_Te_3_ nanoparticles (*κ* = 0.38 W/(m·K)) [2], and Sb_2_Te_3_ electrodeposited films (*κ* = 0.36 W/(m·K)) [3]. In this calculation, we assumed that the ratio of each element (SWCNT film, nanoparticles, and electrodeposited film) in the thermal conductivity was the same as the ratio of the electrical conductivity (See Fig. 4(a)). For the nanocomposite films excluding electrodeposition, the thermal conductivity decreased as the nanoparticle content increased. The thermal conductivity of the nanocomposite film at 100 mg was calculated to be 3.1 W/(m·K). When the electrodeposition was performed, the thermal conductivity was expected to be further decreased. At a nanoparticle content of 100 mg, the thermal conductivity of the nanocomposite film was calculated to be 1.0 W/(m·K) because the large amount of electrodeposition layer with lower thermal conductivity was deposited on the SWCNT surface. In the future, the calculated thermal conductivity should be verified by measuring the thermal conductivity of the nanocomposite films.

**Reference**

[1] Chiba, T., Amma, Y. & Takashiri, M. Heat source free water floating carbon nanotube thermoelectric generators. *Sci. Rep* **11**, 14707 (2021).

[2] Chen, J. et al. Sb_2_Te_3_ nanoparticles with enhanced Seebeck coefficient and low thermal conductivity. *Chem. Mater*. **22**, 3086–3092 (2010).

[3] Yu, Z., Ferrer-Argemi, L., Kim, J., Lim, J.-H., Myung, N. V. & Lee, J. Phase-dependent thermal conductivity of electrodeposited antimony telluride films. *J. Mater. Chem. C* **6**, 3410–3416 (2018).
